# Health-Related Quality of Life Impairment and Indirect Cost of Crohn’s Disease: A Self-Report Study in Poland

**DOI:** 10.1371/journal.pone.0168586

**Published:** 2016-12-16

**Authors:** Przemysław Holko, Paweł Kawalec, Małgorzata Mossakowska, Andrzej Pilc

**Affiliations:** 1 Drug Management Department, Jagiellonian University Medical College, Krakow, Poland; 2 Polish Association Supporting People with Inflammatory Bowel Disease “J-elita”, Warsaw, Poland; 3 International Institute of Molecular and Cell Biology, Warsaw, Poland; 4 Department of Neurobiology, Polish Academy of Sciences, Krakow, Poland; University Hospital Llandough, UNITED KINGDOM

## Abstract

**Background and Aims:**

Evidence on indirect cost of Crohn’s disease (CD) is available but typically provides information on the loss of productivity at paid work of patients. In the present study, the quality of life and indirect costs of CD patients were assessed (overall and by disease severity).

**Methods:**

A self-report questionnaire-based study among adult Polish patients with CD was performed. We collected data on patients’ characteristics, quality of life, loss of productivity, consumption of medical resources, and out-of-pocket expenses. The disease severity was determined using the patient’s version of the Harvey-Bradshaw index. Productivity costs were assessed from the social perspective, using a human capital approach. The cost of absenteeism, presenteeism and permanent work disability was valuated using the gross domestic product per worker. The patients’ productivity loss at unpaid work was measured by time inputs of others to assist patients. The productivity loss among informal caregivers and patients’ productivity loss at unpaid work were valuated with the average wage in Poland. The results were adjusted for confounders.

**Results:**

The responses from 200 patients (47% in remission) were analysed. The mean utility index was 0.839 (SD 0.171). The total indirect cost was estimated at €462.47 per patient per month (24.0%, absenteeism; 35.0%, work disability; 30.4%, presenteeism; 0.4%, productivity loss at unpaid work; and 10.4%, informal care). A significant correlation of the quality of life and productivity losses with disease severity was observed. Compared with active disease, the remission subgroup had a higher utility index by 16% (p<0.001) and lower indirect costs by 71% (p = 0.003) for absenteeism, 41% (p = 0.030) for presenteeism, 76% (p<0.001) for productivity loss at unpaid work, and 75% (p<0.001) for informal care.

**Conclusions:**

Our study revealed the social burden of CD and high dependency of indirect costs and quality of life on the severity of CD in Poland.

## 1. Introduction

Productivity costs usually include the loss of productivity at paid and unpaid work of patients and/or their caregivers, assessed from different economical perspectives such as those of the employer, society, or public finances. Indirect costs of an illness involve mainly temporary or chronic absence from paid work (absenteeism), lower than average workforce productivity during paid work (presenteeism), permanent work disability/unemployment (early departure from the labour market), loss of the patient’s productivity at unpaid work (household work, care work, voluntary work), loss of leisure time, and time inputs of relatives and friends into the care of the patient (productivity loss among informal caregivers) [1,2].

Inflammatory bowel diseases (IBDs) affect people of all ages and constitute a significant burden for the patient and the society [3]. Because of an early onset and chronic character, IBD affects work productivity, with productivity losses resulting from sick leave and work disability amounting up to 50% of the total cost of the disease [4–6]. The high burden in work-related outcomes among patients with IBD have been documented, although only in a limited number of studies assessing productivity loss at paid work due to Crohn’s disease (CD) alone [7,8]. A recent meta-analysis indicated high heterogeneity of available information on productivity cost among patients with IBD, which cannot be overcome by adjustments for macroeconomic indicators[7]. The cost of absenteeism and early departure from the labour market due to IBD varied worldwide from $515 to $14,727 per patient per year (2013 USD), that is, from €32 to €923 per patient per month (€1 = $1.33) [7].

The impact of the severity of CD on the cost of productivity loss at unpaid work and the cost of informal care has not been investigated in detail, and only general information on impairment in usual activities, loss of leisure time, sick leave from paid work among caregivers, or time loss among relatives is available [6,9–13]. The inclusion of informal care can have a strong impact on cost-effectiveness outcomes. It has been recommended to consider the relevance of informal care in the context of economic analysis (e.g., test association with disease severity) and to include it or justify its exclusion [14]; however, limited information on the informal care of patients with CD precludes either.

Although CD is known to have high societal burden, there are sparse data to confirm this for Poland, as there have been no quality of life assessment studies with the calculation of utility indexes and only one unpublished study assessing productivity loss at paid work [15]. The available information suggests that significant between-country differences in health-related quality of life of the same population are possible; the differences may even lead to changing the conclusion of the economic evaluation [16]. In the absence of reliable information on the utility indexes and indirect costs of CD, there is limited ability to highly prioritise its effective treatment and valuate new medical technologies, or determine its optimal treatment using a cost-utilitarian approach. Therefore, we conducted an observational study to assess health-related quality of life and indirect costs of patients with CD (overall and in relation to disease severity).

## 2. Materials and Methods

### 2.1. Study design and participants

This was a self-report questionnaire-based study among adult Polish patients with CD. The inclusion criteria were as follows: proven diagnosis of CD, consent to participate in the study and age of 18 years or older. No exclusion criteria that would preclude participation were used among patients who met the inclusion criteria.

The aim of the study was to obtain information on indirect cost and quality of life in the total sample of the first 200 respondents with CD and the subgroups of patients with disease in remission and those with active disease. The primary aim was to examine whether there is a significant difference in indirect cost between the remission and active-disease subgroups. Other outcomes related to self-reported medical resource utilisation or expenses were also analysed.

### 2.2. Questionnaires

A pack of questionnaires with simple instruction was distributed among patients. The questionnaires were designed to reduce recall bias and evaluate a period of up to a month. For rare events (hospitalisations, specialist consultation, full days of work missed due to CD), the time frame of one month was used in the questions. Other outcomes were measured in a shorter period: five working days for work-related outcomes (presenteeism, single hours of work missed) or a week (impairment in usual activities, regular time inputs from other people to assist the patient). Such approach was used to obtain a similar number of events at different time intervals, and thus to avoid recall bias between the questions. The questions regarding out-of-pocket expenses of patients and financial support applied to the previous month. For questions referring to patients’ characteristics (e.g., age at diagnosis, history of surgical treatment, outcome of the last clinical assessment), there were no time restrictions.

The first part of the questionnaires pack comprised the following elements: general information about the respondent (current age, age at diagnosis, sex, place of residence, comorbidities); information on disease severity, as assessed by a specialist at the last consultation (the respondent indicated disease severity, as communicated to him or her by the specialist at the last consultation); information on surgical treatment of CD in the past (number of surgical procedures since diagnosis and time from the last procedure) and current pharmacotherapy for CD; and information on disease severity, as assessed using the patients’ version of the Harvey-Bradshaw index (P-HBI).

The P-HBI was found to strongly correlate with the specialist-based assessment of the HBI, with a Spearman's *ρ* of 0.82 and the agreement between the clinician and the patient (judging CD as either active or in remission) of 77%, even when the assessment of abdominal mass was excluded [17]. In the present study, the P-HBI questionnaire was complemented with a question regarding abdominal mass (‘present’, ‘absent’, ‘do not know’). Moreover, in addition to standard questions regarding the presence of complications (arthralgia, uveitis, erythema nodosum, aphthous ulcers, pyoderma gangrenosum, anal fissure, fistula, or abscess) in the previous week [17], we included questions regarding the presence of these complications earlier than the previous week in order to obtain additional information about the nature of CD in study participants.

The second part of the questionnaires pack was the three-level, five-dimension questionnaire designed by the EuroQol Group (EQ-5D-3L).

The third part of the questionnaires pack consisted of questions regarding direct cost and resource utilisation associated with CD in the previous month, namely, the total number of consultations with a specialist (publicly funded and private consultations combined), the number of private consultations with a specialist and the average cost of the consultation, the number of hospital stays (one-day hospitalisations included), the length of hospital stays, and the range of out-of-pocket expenses on: i) medications prescribed or recommended by physicians; ii) dietary supplements, special diet, special equipment, transportation to the medical facility; iii) informational materials about the disease, additional hygiene products and others. The patient’s expenses were evaluated using a mixed-answer question with a single-choice question of predefined ranges of expenditures from €0 to €140.8 and an open question if the expenditure was higher.

The last part of the questionnaires pack consisted of questions regarding the loss of productivity at paid work, impairment in usual activities outside the occupational one, informal and formal care, and financial support. The questionnaire was developed with consideration of expert opinions and results of a preliminary internet-based questionnaire study enrolling the sample of 28 anonymous patients with CD. After confrontation with existing tools for the assessment of indirect cost of a disease [1,2,18–20], the questionnaire valid for respondents from Poland was prepared. The questionnaire examined: i) the current patient’s work-related status (pension due to CD, age at transition to a pension, level of inability to work, retirement, retirement age, occupational activity, student status); ii) working time (full time, part time or unspecified with additional questions about the average number of days in a week and hours per day the patient is at work); iii) number of full working days missed due to the disease in the last month; iv) number of single hours of missed work due to CD (e.g., leaving work earlier or going to work later) in the last five working days; v) impact of disease on productivity during paid work in the last five working days (a categorical scale from 0, representing ‘no impact’, to 10, representing ‘completely prevented’); vi) impact of disease on usual activities outside the occupational one in the last week (a categorical scale from 0, representing ‘no impact’, to 10, representing ‘completely prevented’); vii) number of hours and type of assistance the respondent received in the last week due to the disease; and viii) financial assistance or benefits received from government and non-government institutions or relatives due to the disease.

The study procedures were accepted by the representatives of the Polish Association for the Support of People with Inflammatory Bowel Disease “J-elita” and Polish gastroenterologists, whose comments were collected and included in the final version of the questionnaires.

### 2.3. Questionnaire distribution and collection

The questionnaires were distributed through the Association, which has over 1600 members with IBD, grouped in 11 regional branches. Both the members of the Association and unaffiliated patients were intended to be included in the study. Several techniques of dissemination of information about the study were implemented (e-mails, announcement in the magazines published by the Association, at the Association’s forum and web portals, and at events organised by the Association). Around 600 questionnaires were distributed to Polish patients with CD: 400 were sent by e-mail to the randomly selected members of the Association and around 200 were prepared for patients indicating their willingness to participate in the events organised by the Association in all major cities of Poland. The first 200 consecutive questionnaires with at least one answer from patients who met the inclusion criteria were analysed to satisfy sample size criteria. However, the questionnaires from additional 112 patients were received after study completion. These additional questionnaires were included in a sensitivity analysis. The total response rate was estimated at 52%. The collection of the questionnaires was performed in several ways (sending a scanned version to the Association or deposition responses on the web server, submission at the events).

### 2.4. Ethical considerations

No information identifying participants was collected and no answer was required. Participation in the study was voluntary and anonymous. The information about the study including institutions involved, aims and eligibility criteria was available to all patients prior to participation in the description section of the questionnaire and the announcements published by the Association.

The study was conducted without specific approval from an ethics committee as it was not required for the completion of nonintrusive self-reported questionnaires according to Polish law.

### 2.5. Data management and resource valuation

The design of the study eliminated the potential interviewer bias, although imposed the requirement of a more detailed analysis of information obtained from the participants. Each response was strictly evaluated and answers were planned to be assigned as missing when not compatible with other responses (age of diagnosis higher than the current age, absolute reduction of productivity higher than the working time, etc.).

Responses to the P-HBI were analysed according to the instructions provided by Bennebroek Evertsz’ et al. [17], with or without a response to the question about the presence of abdominal mass. Active disease was defined as a P-HBI of more than 4 points. Fistulising CD (penetrating disease course) was identified on the basis of responses to questions about the presence of a fistula (during the previous week as part of the P-HBI, or earlier by means of an additional question).

The responses to the EQ-5D-3L were evaluated with the Polish tariffs [21] to calculate utility indexes and then compare them with age-adjusted and sex-adjusted values for the general population of Poland [22].

The social perspective was adopted and the human capital approach was used to estimate productivity cost at paid work by valuing healthy time lost due to the disease using gross domestic product (GDP) per working hour of a person with occupational activity in Poland in 2015 (GDP of €420,164.45 million divided by a number of employed persons, that is, 16,234 thousand and by the number of maximum working hours in 2015, that is, 2016) [23–25]. The approach can be perceived as the loss of an investment in a person’s human capital, but recognising that only part of the population has an occupational activity, working time is not the only factor of production and reduced performance or absence of an employee also prevents the use of complementary factors of production (quality of the workforce). The conventional mean value of output elasticity of labour according to the Cobb-Douglas function of production (0.65) [26] was implemented to estimate the final unit cost of productivity loss at paid work of €8.34 per hour.

The reduction of productivity at paid work due to absenteeism, R_A_, was calculated from the formula:

$$R_{A}=\frac{A_{d}\cdot L_{h}+\frac{A_{h}}{5}\cdot \left(L_{d}-A_{d}\right)}{L_{d}\cdot L_{h}}$$

Where A_d_ is the number of full days missed from paid work due to CD in the previous month; A_h_ is the number of single hours missed from paid work due to CD in the last five working days; L_d_ is the patient’s maximum working time per month expressed as working days in a month; and L_h_ is the patient’s maximum working time per month expressed as working hours per day. The reduction of productivity at paid work due to presenteeism, R_P_, was calculated by multiplying by a factor of 0.1 the patients’ response to the question regarding the impact of CD on productivity during paid work in the last five working days (presenteeism score). The overall reduction of productivity at paid work, R_O_ (absenteeism and presenteeism combined), was calculated from the formula: R_O_ = R_A_ + (1 –R_A_)·R_P_.

The hours missed from paid work per month due to absenteeism and presenteeism were calculated for each patient by multiplying the number of working hours in a month (L_d_·L_h_) by R_A_ and (1 –R_A_)·R_P_ for absenteeism and presenteeism, respectively. The indirect cost of CD was defined as the product of hours missed from paid work and the unit cost of productivity loss.

The productivity cost of early departure from the labour market was calculated using the prevalence method applied in cost-of-illness studies[27]. No adjustment for the friction period was made and only registered departures from the labour market were considered (i.e., among patients with registered full or partial inability to work, on social or disability pension). The approach measures the value of productivity lost during a specified period of time, irrespective of the time of departure from the labour market. For example, a patient at a productive age, being on disability pension for two years, with registered 75% inability to work, generated the cost of early departure from the labour market at the product of the level of disability, the number of working hours in Poland in the previous month (168 hours on average) and the unit cost of productivity loss at paid work. The social pension in Poland is granted to patients with full inability to work that occurred before the age of 18 years or during learning, so the 100% inability to work was assumed for patients who received social pension.

There are two main approaches to measuring productivity loss at unpaid work used in the literature: 1) asking respondents about the amount of time a substitute would need to perform activities they were not able to perform themselves due to the disease; and 2) asking respondents about the amount of time during which they actually received help with unpaid work due to the disease [1]. The costs of informal care are mainly related to the time inputs of informal caregivers. The time inputs are measured by asking patients or (usually) caregivers about the amount of time spent on care [14,28–30]. To valuate unpaid work and informal care, a shadow price is assigned to the missed time dedicated for activities related to them. The proxy good approach and opportunity cost approach are the most popular ones in the literature. The value of lost unpaid work or informal care is determined using the value of the closest market substitute or a respondent’s value of competing time use (usually the value of paid work), respectively [1,2,20]. Alternative valuation methods were also proposed to valuate informal care [28–30].

In our study, the productivity loss at unpaid work of patients was measured with the inclusion only of time with any assistance (of relatives, nurse, social assistant, paid assistant) required due to CD, that is, only replaced unpaid work was included. Such approach was used to exclude any patients’ activities that can be affected by illness but that are not urgent and can be done later without any significant output of unpaid work loss [1,2]. The productivity loss among informal caregivers (informal care) was measured with the inclusion of the total time spent on the care of a participant, using the recall method [29]. The opportunity cost method [2,28–30] was used to value unpaid work and informal care with unit cost at an average wage per hour of work in Poland, that is, €5.27 (the average monthly wage in the third quarter of 2015 divided by the maximum number of working hours in the period) [31]. The product of the number of hours of assistance in the previous week and the unit cost of productivity loss was multiplied by the number of weeks in the previous month (4.345 on average) to obtain monthly estimates of informal care cost or the cost of productivity lost at unpaid work, depending on the type of care used in the calculation.

The costs of publicly funded consultations, hospitalisations and treatment indicated by respondents were assessed via official remuneration schemes. The official sources of information on unit costs were used. The lowest cost of a specialist consultation in Poland was used in the calculation (W11 code, first-type consultation with a gastroenterologist, which includes basic examinations [32]). The one-day hospitalisation and diagnostic examinations during a biological treatment were obtained from the ordinance of the President of the National Health Fund [33]. The cost of surgical hospitalisation was derived using the diagnosis-related group (DRG) system. We used the average cost of DRG-codes F51 (‘complex procedures in inflammatory bowel diseases’) and F52 (‘large and endoscopic procedures in inflammatory bowel diseases’), weighted by the number of occurrences in 2014 in Poland. For medical hospitalisations, we used the most frequently billed DRG-code among patients with IBD in Poland (F58, ‘inflammatory bowel diseases’) [34].

A daily dose defined by the World Health Organization [35] was used to obtain average drug utilisation among study participants. Mercaptopurine was assumed to be administered at a daily dose of 1.25 mg per kg of body weight. All medicines were priced using the weighted average cost of unit packages refunded in 2015 in Poland. All packages’ sizes and all products reimbursed in Poland were considered in the calculation [36]. The average body weight among patients treated with biological agents in the years 2010–2012 in Poland (64.71 kg [37]) was used for the assessment of utilisation of medicines with the dosage depending on body weight. In Poland, biological treatment is administered for a period of no longer than 12 months [33]. The information on the duration of treatment was not collected in the study, and the average monthly cost of adalimumab and infliximab treatments was used (the total cost of the 12-month therapy with recommended dosing divided by 12). The unit costs are presented in Table 1.

**Table 1:** Direct medical unit costs from the perspective of public payer in Poland.

Category (unit)	Unit cost	Sources
Consultation with specialist	€7.57	[32]
One-day hospitalisation	€109.80	[33]
Medical hospitalisation	€975.98	[34]
Surgical hospitalisation	€1553.16	[34]
Sulfasalazine (2 g)	€0.53	[35,36]
Mesalazine (1.5 g)	€0.53	[35,36]
Prednisone (10 mg), prednisolone (10 mg) or methylprednisolone (20 mg)	€0.24	[35,36]
Budesonide (9 mg)	€0.03	[35,36]
Azathioprine (0.15 g)	€0.37	[35,36]
Mercaptopurine (80.89 mg)	€0.31	[36,37] and assumption
Methotrexate (2.5 mg)	€0.77	[35,36]
Adalimumab (80 mg)	€967.01	[36]
Infliximab (100 mg)	€298.23	[36]
Diagnostics during biological treatment (month of treatment)	€54.90	[33]

The valuation of self-reported out-of-pocket expenses was made with an average, minimal and maximal value from the range indicated by a respondent to access the measurement error. The results of an average valuation with the maximal relative measurement uncertainty obtained from the results of extreme valuation methods were presented in the manuscript.

To enhance generalisability, some cost outcomes were presented as a share of the average monthly GDP per capita in Poland in 2015, that is, an annual value of €10,900, which equals 68% of the average GDP per capita in the European Union [38], divided by 12.

All cost outcomes were calculated in PLN and converted to Euros with the exchange rate of 4.2624 PLN per €1, that is, the average rate in the fourth quarter of 2015 [39], the period of which cost data applied.

### 2.6. Statistical analysis

The sample size was derived using a power of 80%, the level of significance of 5%, and the ability to detect a €200 difference in the total indirect cost (early departure from the labour market excluded) between the subgroups assuming unequal variances (variance in the remission subgroup reduced by half), 10% of missing responses and an equal recruitment ratio.

All the study outcomes and patients’ characteristics were analysed descriptively and presented as a mean with standard deviation (SD) or median with interquartile range (IQR) for continuous variable and frequencies for categorical variables.

Correlations were assessed by the Spearman's ρ rank correlation coefficient. The Pearson χ^2^ test for categorical variables and the Wilcoxon rank-sum test for continuous variables were applied for comparison of the subgroups. The Wilcoxon signed-rank test was applied for comparison between utility indexes of participants and general population.

The concordance between current disease activity and disease activity assessed by a specialist at the last consultation was assessed by the percentage agreement and Cohen’s κ.

The fractional logit regression was used to obtain adjusted means and confidence intervals (CIs) for utility indexes in the subgroups. Due to skewed data distribution and large mass of observations with zero-costs, two-part models (*twopm* command [40]) with robust estimator of variances were used to analyse differences in indirect costs between the subgroups [41]. All models included a binary variable indicating disease activity (remission or active disease) and covariates (sex, age, age at diagnosis, presence of comorbidities, surgical treatment before enrolment to the study, pension) to control for possible confounders. Model selection and assessment were based on the Box-Cox test, modified Park test and (pseudo)log-likelihood. Average adjusted predictions and marginal effects were presented as adjusted means with 95% CIs calculated using the delta method.

Selected outcomes were presented on box-whisker diagrams with the box representing IQR; transversal line, median; the ends of the whiskers, 5 and 95 percentiles; ‘x’ marks, minimum or maximum; and dark bullets, average values.

The analyses included all questionnaires with at least one answer. Missing data were excluded from the analysis of an outcome. No correction for multiplicity was implemented; a p value of less than 0.05 was considered statistically significant.

Reporting of the study was done in adherence with the Strengthening the Reporting of Observational Studies in Epidemiology Statement [42].

Data preparation and statistical analysis were performed in Excel 2016 (Microsoft Co., Redmond, WA) and STATA 13 (StataCorp, College Station, TX). Figures were prepared in OriginPro 8 (OriginLab, Northampton, MA).

The patient-level data described in the manuscript are available as S1 Dataset.

## 3. Results

The questionnaires from 207 patients were collected between November 14 and December 7, 2015. Seven patients were excluded because they did not meet the inclusion criteria (all excluded patients at the age of <18 years).

The comparison of current disease activity and disease activity assessed by a clinician at the last consultation revealed a percentage agreement of 79.1% and Cohen’s κ of 0.497 (p<0.001, n = 193). The current disease status was not assessed for two patients, and they were excluded from the subgroup analysis. Patients’ characteristics were similar between the subgroups, with the exception of a higher use of glucocorticoids and a higher rate of patients on a pension among those with active disease (Table 2 and S1 Table).

**Table 2:** Characteristics of study participants (overall and in relation to disease severity).

Characteristic	Remission (N = 93)	Active disease (N = 105)	All patients (N = 200)	P value between subgroups
Age, mean (SD)	30.60 (9.94)	32.86 (10.75)	31.80 (10.41)	0.087
Male gender, n (%)	40 (43.0)	44 (41.9)	84 (42.2)	0.875
Age at diagnosis, mean (SD)	24.14 (10.26)	24.73 (10.88)	24.46 (10.57)	0.548
Comorbidities, n (%)	35 (37.6)	50 (47.6)	87 (43.5)	0.157
*Current treatment*, *n (%)*				
Mesalazine or sulfasalazine	75 (80.6)	90 (85.7)	166 (83.0)	0.339
Azathioprine, mercaptopurine or methotrexate	52 (55.9)	50 (47.6)	104 (52.0)	0.244
Budesonide, prednisone, prednisolone or methylprednisolone	13 (14.0)	37 (35.2)	51 (25.5)	**0.001**
Infliximab or adalimumab	10 (10.8)	18 (17.1)	28 (14.0)	0.198
History of surgical treatment, n (%)	38 (40.9)	53 (51.5)	92 (46.5)	0.138
Months since the last surgery for CD among patients with a history of surgery, median (IQR)	33 (12 to 70)	27 (10.5 to 55)	30 (12 to 60)	0.781
Penetrating disease course, n (%)	19 (20.4)	34 (33.3)	54 (27.4)	0.127
*Disease activity at the last clinical assessment*, *as indicated by the respondent*, *n (%)*				
Remission or mild disease	84 (90.3)	57 (54.3)	141 (70.5)	**<0.001**
Moderate or severe disease	6 (6.5)	44 (41.9)	52 (26.0)	
'don't know'/ ‘don’t remember’	3 (3.2)	4 (3.8)	7 (3.5)	
Current disease activity, mean P-HBI (SD)	1.88 (1.37)	9.20 (3.98)	5.76 (4.76)	not applicable
*Work status*, *n (%)*				
Any occupational activity	60 (64.5)	58 (55.2)	120 (60.0)	0.184
Retired	2 (2.2)	2 (1.9)	4 (2.0)	0.902
On a social or disability pension	4 (4.3)	16 (15.2)	21 (10.5)	**0.011**
Student	21 (22.6)	21 (20.0)	42 (21.0)	0.658

### 3.1. Health-related quality of life

The ‘anxiety’ and ‘pain’ domains of the EQ-5D questionnaire were the most affected domains by CD, especially among patients with active disease (Table 3).

**Table 3:** Frequency of responses to the EQ-5D-3L questionnaire. Data presented as number of respondents with percentage in brackets.

EQ domain	Study subgroup	No problems	Some problems	Severe problems
Mobility	All patients	166 (83.4%)	33 (16.6%)	0 (0%)
	Remission	92 (98.9%)	1 (1.1%)	0 (0%)
	Active disease	73 (70.2%)	31 (29.8%)	0 (0%)
Self-care	All patients	193 (97.0%)	6 (3.0%)	0 (0%)
	Remission	92 (98.9%)	1 (1.1%)	0 (0%)
	Active disease	100 (96.2%)	4 (3.8%)	0 (0%)
Usual activities	All patients	128 (64.3%)	68 (34.2%)	3 (1.5%)
	Remission	75 (81.5%)	17 (18.5%)	0 (0%)
	Active disease	53 (50%)	50 (47.2%)	3 (2.8%)
Pain	All patients	61 (30.5%)	128 (64.0%)	11 (5.5%)
	Remission	48 (51.6%)	44 (47.3%)	1 (1.1%)
	Active disease	13 (12.4%)	83 (79%)	9 (8.6%)
Anxiety	All patients	72 (36.0%)	112 (56.0%)	16 (8.0%)
	Remission	42 (45.2%)	51 (54.8%)	0 (0%)
	Active disease	30 (28.6%)	59 (56.2%)	16 (15.2%)

**Fig 1:**
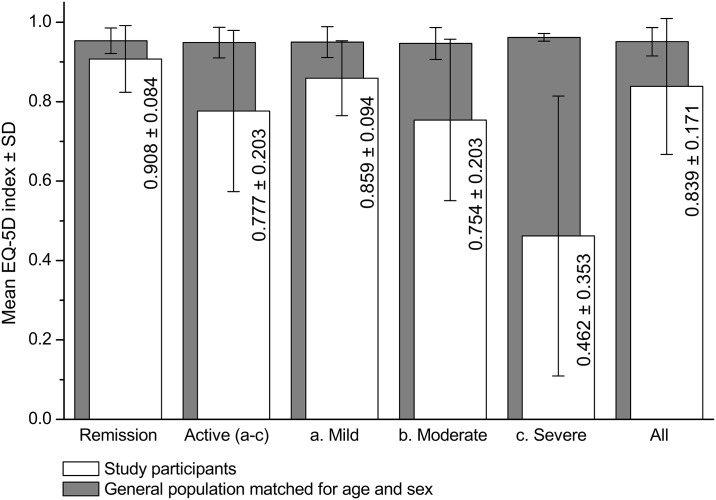
Unadjusted utility indexes among study participants and members of general population matched for age and sex. p<0.001 for the comparison of utility indexes between the remission and active-disease subgroups and comparison between study participants and members of the general Polish population matched for age and sex.

Utility indexes among study participants were significantly lower than the corresponding values in general population matched for age and sex (Fig 1), and were correlated with the P-HBI (ρ of -0.586, p<0.001).

The adjusted mean utility index in the subgroup with active disease was estimated at 0.784 (95% CI: 0.748 to 0.820). The remission subgroup had a higher utility index by 0.124 (95% CI: 0.082 to 0.165, p<0.001).

### 3.2. Absenteeism and presenteeism

The productivity at paid work was strongly impaired by CD among study participants. Working patients missed on average 22.37 hours of paid work per month (SD 42.24 hours) and had a reduced quality of production during paid work, resulting in a mean absenteeism cost of €186.66 (SD €352.45) and presenteeism cost of €235.09 (SD €250.40) per month per patient with occupational activity. The reduction of work productivity was moderately but significantly correlated with the P-HBI, with a Spearman's ρ of 0.301 (p = 0.001) for absenteeism, 0.487 (p<0.001) for presenteeism and 0.494 (p<0.001) for both.

**Fig 2:**
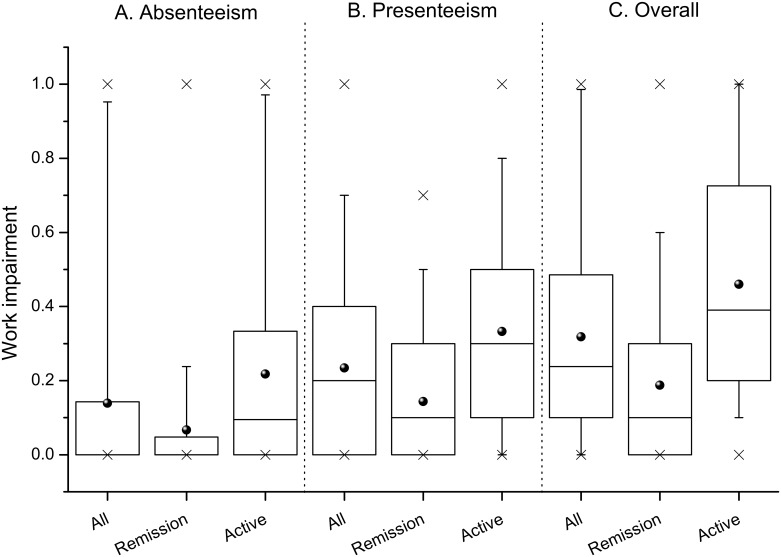
Impairment in productivity at paid work among study participants with occupational activity. Box diagrams of the reduction of productivity at paid work due to: (A) absenteeism; (B) presenteeism; and (C) both (overall and in relation to disease severity); p<0.001 for the comparison between the remission and active-disease subgroups for all outcomes.

Work impairment differed significantly between the subgroups (Fig 2), resulting in a lower adjusted cost of absenteeism by 71% (€123.20 per month per patient, 95% CI: 42.77 to 203.62, p = 0.003) and presenteeism by 41% (€70.59, 95% CI: 6.97 to 134.22, p = 0.030) among participants with disease in remission (Table 4).

**Table 4:** Monthly indirect costs of Crohn’s disease by disease severity.

	Remission	Active disease
*I*. *Absenteeism*		
Unadjusted mean (SD), n = 195	€59.61 (210.42)	€157.43 (336.61)
Adjusted mean (95% CI) ^a^, n = 192	€51.00 (19.76 to 82.25)	€174.20 (97.09 to 251.31)
*II*. *Presenteeism*		
Unadjusted mean (SD), n = 195	€102.10 (183.52)	€169.51 (249.12)
Adjusted mean (95% CI) ^a^, n = 192	€100.24 (62.51 to 137.96)	€170.83 (120.71 to 220.96)
*III*. *Productivity loss at unpaid work*		
Unadjusted mean (SD), n = 194	€18.88 (64.40)	€77.89 (143.44)
Adjusted mean (95% CI) ^a^, n = 191	€18.74 (6.35 to 31.14)	€78.86 (50.45 to 107.27)
*IV*. *Informal care*		
Unadjusted mean (SD), n = 194	€18.88 (64.40)	€74.68 (137.39)
Adjusted mean (95% CI) ^a^, n = 191	€18.96 (6.38 to 31.54)	€75.41 (48.24 to 102.60)
*All (I + II + III* ^b^ *+ IV)*		
Unadjusted mean (SD), n = 192	€178.43 (282.88)	€406.05 (468.93)
Adjusted mean (95% CI) ^a^, n = 189	€173.08 (118.51 to 227.65)	€416.52 (318.49 to 514.54)

^a^ adjusted for age, age at diagnosis, comorbidities and surgical treatment.

^b^ patients’ productivity loss at unpaid work, that was compensated by informal caregivers were excluded from the calculation.

### 3.3. Usual activity impairment and informal care

**Fig 3:**
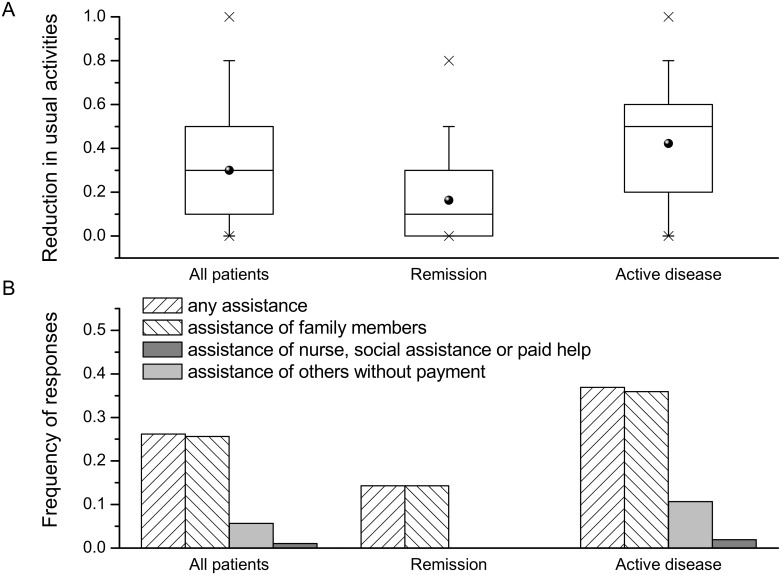
Impairment in usual activities outside the occupational activity among study participants. (A) Box diagrams of impairment in usual activities (overall and in relation to disease severity); (B) the frequency of participants that required assistance in their usual activities (overall and in relation to disease severity); p<0.001 for the comparison between remission and active-disease subgroups for both outcomes.

More than three-fourth of the participants indicated a reduction of their usual activities due to CD. Around one-fourth of the patients required assistance in performing usual activities, predominantly of their relatives (Fig 3). Family members and non-relatives without payment dedicated an average of 8.15 (SD 6.62) and 2.80 (SD 1.40) hours per week, respectively. The time inputs from other persons amounted to 37.11 (SD 29.11) hours per month per patient with assistance and overall 9.70 (22.04) hours per month.

The mean cost of productivity loss at unpaid work and informal care was estimated at €49.95 (SD €116.71) and €48.26 (SD €112.41) per month, respectively.

Impairment in usual activities was correlated with the P-HBI (ρ of 0.593, p<0.001) and the rate of assistance (ρ of 0.501, p<0.001). Impairment in usual activities and the rate of assistance significantly differed between the subgroups (Fig 3), resulting in a lower adjusted cost of productivity at unpaid work and informal care in the remission subgroup by 76% (€60.11 per month per patient, 95% CI: 28.80 to 91.43, p<0.001) and 75% (€56.46 per month per patient, 95% CI: 26.28 to 86.64, p<0.001), respectively (Table 4).

### 3.4. Total indirect cost

Around 13% of the participants were on social or disability pension. The median level of incapacity to work was 100% (IQR: 50% to 100%). The mean cost of registered early departure from the labour market (permanent work disability) was estimated among those participants at €1227.77 per month (SD €344.96).

The components of the indirect cost of CD per adult patient are presented in part I of Table 5. The total indirect cost amounted to €462.47 per patient per month, that is, 50.9% of GDP per capita. Absenteeism, early departures from the labour market, presenteeism, productivity loss at unpaid work uncompensated by informal caregivers and informal care amounted to 24.0%, 35.0%, 30.2%, 0.4%, and 10.4% of the total indirect cost, respectively.

**Table 5:** Monthly costs of Crohn’s disease in Poland.

Category, number of respondents	Mean cost per respondent (SD)
*I*. *Indirect costs*	
Absenteeism, n = 197	€110.86 (286.29)
Registered early departure from labour market, n = 197	€162.04 (434.45)
Presenteeism, n = 197	€139.62 (224.73)
Productivity loss at unpaid work, n = 195	€49.95 (116.71)
Informal care, n = 195	€48.26 (112.41)
*II*. *Direct costs*	
Publicly funded consultation with specialist, n = 196	€6.62 (9.25)
Private consultation with specialist, n = 196	€16.62 (30.99)
Hospitalisations—public payer’s perspective, n = 194	€202.11 (496.82)
Current treatment—public payer’s perspective, n = 200	€173.31 (382.31)
All medications prescribed or recommended by physicians (patients’ perspective), n = 197	€45.25 (36.98) ^a^
Dietary supplements, special diet, special equipment, transportation to the medical facility (patients’ perspective), n = 193	€34.64 (35.70) ^b^
Informational materials, additional hygienic articles and others (patients’ perspective), n = 194	€13.97 (8.93) ^c^
*III*. *Transfer costs*	
Social benefits or financial support from public organisations, n = 196	€39.73 (88.19)
Financial support from others, n = 195	€14.83 (50.46)

^a^ the maximal relative measurement uncertainty ranged from -37.8% to 20.8% of presented value;

^b^ as above, from -43.2% to 30.9%;

^c^ as above, from -85.2% to 82.7%

Adjusted total indirect cost (early departure from the labour market excluded) among participants with disease in remission was reduced by 60% compared with the subgroup with active disease, that is, €243.44 per patient per month (95% CI: 131.49 to 355.39, p<0.001; Table 4).

### 3.5. Direct and transfer costs

Around 70% of the patients (138 of 196) had consulted their disease status with a specialist in the previous month. The mean number of consultations was 1.87 (SD 1.30), of which 0.63 (SD 0.75) were financed by patients at a cost of €36.61 (SD €15.94). Forty-eight (24.7%) patients were hospitalised due to CD in the month preceding enrolment, with a median number of hospitalisations of one (IQR: 1 to 2) and a total length of stay at hospital of three days (IQR: 1 to 8.5; S1 Fig). Surgeries were performed in 5 out of 48 patients hospitalised at least once in the previous month; surgeries accounted for 6.25% of all hospitalisations (one-day hospitalisations included).

Patients indicated higher expenses on medicines than in the case of other categories. Around one-third of the patients indicated expenses on medicines as less than €23.5, from €23.5 to €46.9, or from €46.9 to €93.8 monthly. More than 52% of the patients indicated expenses on dietary supplements, special diet, special equipment and transportation to the medical facility as being less than €23.5 monthly. Further expenses were rated by 92.8% of patients as being lower than €23.5 monthly (S2 Fig).

Forty-six patients (23.4%) received financial support from public organisations (11, social pension; 15, pension for incapacity to work; 8, rehabilitation benefits; 20, nursing benefits; 8, others). Thirty-seven patients (19%) regularly received financial support from relatives and/or non-profit organisations and 13 patients (6.7%) received support from both sources.

The components of direct and transfer costs of CD per adult patient are presented in parts II and III of Table 5.

Post hoc analyses indicated that there were significant differences between remission and active-disease subgroups in terms of the total number of consultations in the previous month (p<0.001), monthly cost of consultations from the public payer’s perspective (p = 0.004), cost of current treatment from the public payer’s perspective (p = 0.014), amount of benefits received from public organisations (p = 0.017), and self-reported expenses on all categories (p value: 0.002 to 0.024).

## 4. Discussion

So far there have been a limited number of studies assessing indirect costs of CD, with sparse information on the outcomes in Poland, no information on utility indexes of Polish patients with CD and lack of information on the informal care of patients with CD in relation to disease severity, which provided the grounds for conducting the current study.

Nearly a half of the participants were in remission. The comparison of the P-HBI results and disease activity assessed by a clinician at the last consultation indicated a moderate agreement. However, 30% of the patients did not consult their disease status in a month preceding enrolment, during which the change of disease severity might have occurred. The limitation of the patients’ version of disease severity assessment might have also affected the comparison [17]. The total cost of CD from the societal perspective was estimated at around €1000 per month per adult patient (110% of the GDP per capita), with an indirect cost amounting to 51% of the total cost. However, each component of the total cost was assessed with varied precision (e.g., a less precise assessment of direct costs, particularly the self-reported expenses of the patients), which might have influenced the precision of the total cost and the share of indirect cost assessment.

The comparison of the outcomes between the subgroups indicated high impact of disease severity on the health-related quality of life and short-term indirect costs. The cost of informal care constituted the lowest share of the total indirect cost of CD, but was characterised by a strong association with disease severity. The results of the study suggest that the induction and maintenance of remission in all participants with active disease might reduce the total indirect cost of CD by at least 41% (when no impact of current disease severity on the early departures from the labour market is assumed), thus ensuring an argument to highly prioritise effective CD treatment, even under restricted budgetary conditions. Nevertheless, since data were collected once for each participant, the relationship between the outcomes and disease severity may require additional confirmation in a longitudinal survey.

The study was not designed to assess the difference in cost of early departure from the labour market between the subgroups, but a higher proportion of patients with active disease were on social or disability pension than that of patients with disease in remission. It might be explained by the proportion of patients with continuously active disease, who therefore had higher odds of qualifying for disability pension. However, the post hoc analysis indicated that the different rate of being on pension did not translate into a significant difference in cost of permanent work disability (a marginal cost of €81.61, 95% CI: -43.73 to 206.96, p = 0.202).

The response rate (52%) was considered sufficient. However, there are several reasons to believe that the real response rate could not be determined. First of all, the Association has no information about the character of the disease in a large proportion of members, and the number of effectively distributed e-mails (i.e., those not designated as spam) was unknown. Secondly, the members of the Association could complete the questionnaires that were distributed at the events, and the questionnaires received via e-mail might have been shared with other patients.

The strengths of this study included both the size and the diversity of participants, obtained by direct enrolment without additional restrictions (e.g., at consultation with a specialist, during hospitalisation). An important limitation is the possible sampling bias. The sample was not necessarily representative of the CD population as a whole. In our sample, we observed the likely overrepresentation of women and patients living in cities with at least 100 thousand inhabitants. The proportion of fistulising CD and patients with a history of surgical treatment is another possible validation of representativeness. However, the characteristics of the study sample reflected the recent trends observed in Europe, namely, the presence of a fistulising disease in up to one-third of patients with CD and a history of surgical treatment—in up to 50% [43]. The rate of previous surgical treatment for CD among our study participants was lower or comparable to that in other European studies on indirect cost (70% of German patients [5], 41% of Hungarian patients [44] and 54% of Dutch patients [6]).

Other limitations of the study are related to the design as self-reported information can be influenced by, for example, recall, response or social desirability biases. The patients’ responses were not validated with medical records or during a clinical examination.

Several sensitivity analyses were conducted (data not shown). The different unit cost of productivity losses at paid work resulted in a relative change of the total indirect cost by: -28% (unit cost at the GDP per capita), -30% (average salary) or 29% (gross value added per worker). The exclusion of abdominal mass from the P-HBI did not significantly change the classification of participants into the subgroups or the conclusions. The inclusion of the additional questionnaires received after study completion did not affect the conclusions. The results were slightly different. The differences between the study subgroups in utility weights and in total indirect cost, obtained from statistical models, were estimated with combined dataset at 0.114 (-8% compared with the study sample) and €318.74 (+9%), respectively. The human capital approach is sometimes considered to overestimate indirect costs, and the frictional cost approach is then preferable [2,19]. We found that the incorporation of a conventional friction period (90 days) did not influence the majority of conclusions. The short-term indirect costs did not change, but the cost of early departures from the labour market was fully reduced because all departures had occurred in a period of more than 90 days before enrolment.

The adjustment for multiplicity was not incorporated. It is possible that the incorporation of the conservative Bonferroni correction (threshold at p = 0.0033) would not change general conclusions, although the differences between the subgroups in the rate of pension and the cost of presenteeism would no longer be significant, and all results would have lower statistical power.

The study results are in line with earlier findings. In a systematic review, Kawalec and Malinowski [7] indicated that only 18 studies on the indirect costs of IBD were conducted worldwide: six studies on CD, one on ulcerative colitis and eleven on IBD in general. All identified studies were mainly related to the loss of productivity at paid work, but only two assessed presenteeism. The review indicated that an adult patient with IBD generated a mean annual indirect cost (absenteeism and early departure from the labour market only) of $6433 (95% CI: $280 to $12,586), corresponding to a monthly estimate of €403 (95% CI: 18 to 789), which is higher by 32% than that in our study (€272.90).

Mandel et al. [44] showed that the average cost of productivity loss due to work disability (early departure from the labour market), absenteeism and presenteeism among Hungarian patients with CD was €1545, €395 and €2605 per patient per year, respectively. After conversion to monthly estimates, the cost of early departure from the labour market and absenteeism was lower than that in our sample, by 21% and 70%, respectively, but the cost of presenteeism was higher by 55%. The difference in patients’ characteristics (e.g., biological treatment administered in 31% of Hungarian patients) and the difference in the unit cost of productivity loss (€5.96 vs. €8.34) were presumably the main reasons for these discrepancies. In a sample of 241 German patients with CD (mean age of 41 years, 35% of men, 47% of patients in remission, 70% of patients after surgical treatment for CD), the cost of absenteeism and disability due to CD amounted to €218 and €700 per patient per four weeks, respectively [5]. The estimates were at least two times higher than in our sample, which can be partially explained by the difference in unit costs used in both studies (from €14.28 to €35.71, depending on age and sex [5] vs. €8.34). van der Walk et al. [6] estimated the cost of absenteeism, productivity lost at unpaid work and sick leave of paid work among caregivers of Dutch patients with CD at €288.57, €19.40 and €17.76 per patient per three months, respectively. The monthly estimates calculated from those values were lower than those in our sample, by 13%, 88% and 87%, respectively. The difference in patients’ characteristics (79% of Dutch patients with CD in remission; 23% treated with biological agents) and the definition of productivity losses (unpaid work constituted voluntary work only; among caregivers—sick leave from paid work only) were presumably the main reasons for these discrepancies. The indirect cost among Spanish patients with CD was estimated at €4704 (95% CI: €4335 to €5073) per patient per year, that is, €392 (95% CI: €361 to €423) per month. However, only absenteeism among patients with occupational activity and loss of leisure time among unemployed patients were included in the study [4]. Mesterton et al. [45] estimated the indirect cost of CD in Sweden at €465 per patient per four weeks (absenteeism and presenteeism), which was nearly two times higher than that in our study (€250.48).

Wladysiuk et al. [15] reported that among CD patients with occupational activity from Poland the mean reduction of productivity at paid work due to absenteeism, presenteeism and both equalled to 16%, 24% and 36%, respectively. The annualised cost of absenteeism and presenteeism among participants with any occupational activity was estimated at €2393 and €3151, respectively, and was slightly higher than the results of the current study (annualised estimates of €2240 and €2821, respectively). The discrepancies might be related to the differences in patients’ characteristics (remission among 38% vs. 47%, average age at 36 vs. 31.8 years, frequency of males at 52% vs. 42.2%) or the enrolment procedure (at consultation with a specialist vs. invitation by the Association). In particular, we found that among participants with occupational activity who consulted their disease status with a specialist during the month prior to enrolment, the mean cost of absenteeism and presenteeism was higher compared with the total sample, by 28% and 6%, respectively.

The reduction of work productivity due to absenteeism and presenteeism ranged from 16% to 36% and from 40% to 58%, respectively, among patients with active disease enrolled to multinational trials [10,46]. The overall work impairment equalled to 27% among CD patients from Spain [12]. The impairment of the usual activity among CD patients was comparable with earlier findings as well: 35% among CD patients from Spain [12] and 51% among patients with active disease [10]. We observed that the non-monetary outcomes of our study were compatible with other reports, which confirms the credibility of the study design.

The correlation between disease severity and productivity impairment at paid work or the quality of life were demonstrated [4,11,12,44,45]. The impact of disease severity on informal care was not assessed elsewhere, although Benedini et al. [9] reported a correlation between the cost of relatives’ time loss with the quality of life among Italian patients with CD.

In a longitudinal study of patients from eight European countries and Israel, Odes et al. [47] demonstrated the mean annual cost of medications of €663 and hospitalisation cost (medical and surgery hospitalisations combined) of €1606. The values converted to monthly estimates were lower than those observed for Polish patients, by 34% and 64%, respectively. However, the authors noted that biological treatment was uncommon in their sample and the difference in the rate of hospitalisations was the primary cause of substantial between-country variations in the direct cost. In our study 14% of patients were treated with biological agents. The hospitalisation cost among Polish patients was comparable to that among patients from Portugal (€2074 annually) and Denmark (€2676) enrolled to the study by Odes et al. A higher rate of hospitalisations in Poland compared with other countries from the study by Odes et al. was confirmed by Vegh et al., who demonstrated a significantly higher hospitalisation rate and a significantly lower rate of biological treatment in CD patients in Eastern Europe than in Western Europe or Australia [48].

van der Valk et al. [6] obtained direct costs of CD in the Netherlands. The rate of outpatient consultations was higher by 64%; the rate of hospitalisations was lower by 46%; and the rate of medication use was higher by 120% among patients in the Netherlands compared with our sample. Again, the between-country differences in the rate of hospitalisations, more frequent administration of biological treatment and a higher percentage of Dutch patients with CD in remission than in our sample were presumably the reasons for the discrepant results of our studies.

The high rate of hospitalisations observed in our sample may be explained by the organisation of the health care system in Poland. Some medical and diagnostic procedures (e.g., administration of a biological agent and assessment of its efficacy) are performed during short hospital stays only.

The results of our study indicated high relevance of indirect cost (including informal care) in the context of economic analyses and can be used to inform economic models created to assess the cost-effectiveness of new treatment or to assess optimal treatment sequence.

## Supporting Information

S1 Dataset Data obtained from 200 study respondents. (XLSX)

S1 Fig The number of consultations with specialist and hospital stays in a month preceding enrolment on the study. (PDF)

S2 Fig The frequencies of reported monthly patients’ out-of-pocket expenses. (A) Medications prescribed or recommended by physicians; (B) dietary supplements, special diet, special equipment, transportation to the medical facility; (C) informational materials about the disease, additional hygiene products and others.
(PDF)

S1 Table Additional characteristics of the study participants, overall and in relation to disease severity. (PDF)
