# Supplementary figures and images for: Health-Related Quality of Life Impairment and Indirect Cost of Crohn’s Disease: A Self-Report Study in Poland
Source: PLoS One. 2016 Dec 16;11(12):e0168586. doi: 10.1371/journal.pone.0168586 (PMC5161376; doi:10.1371/journal.pone.0168586)

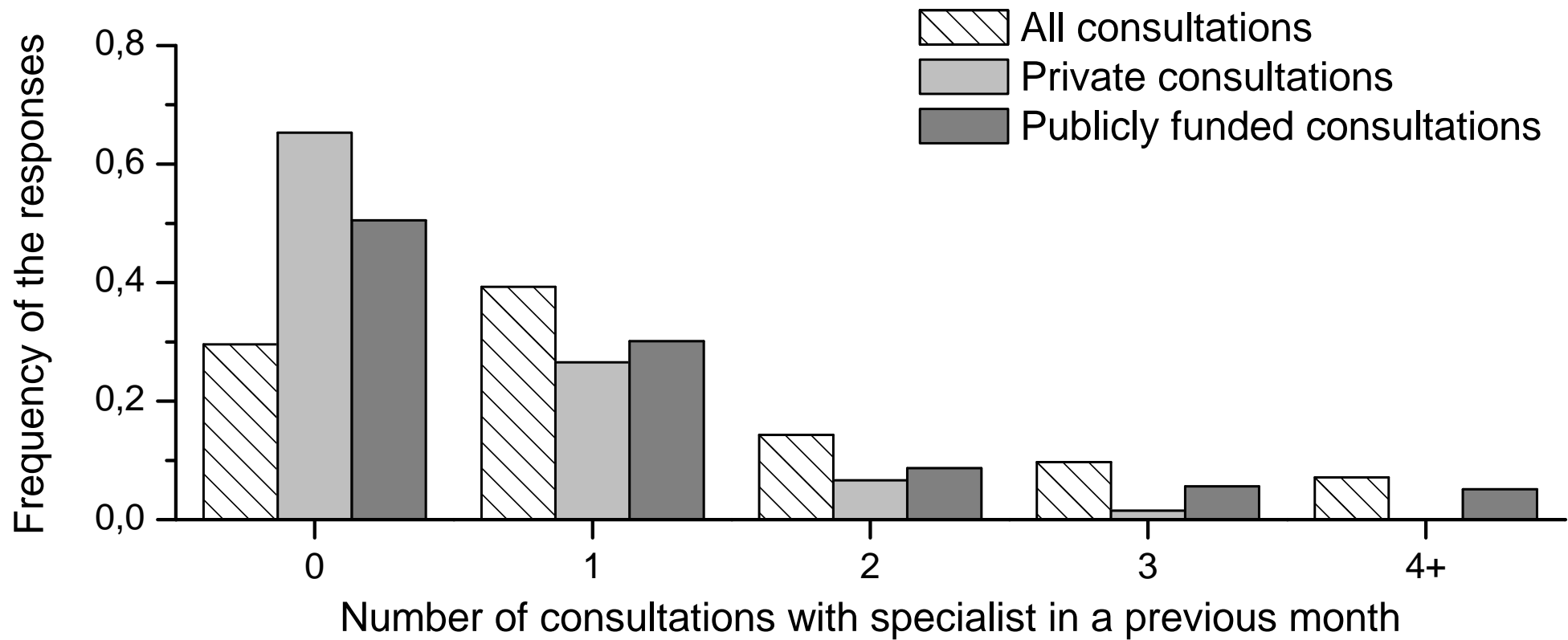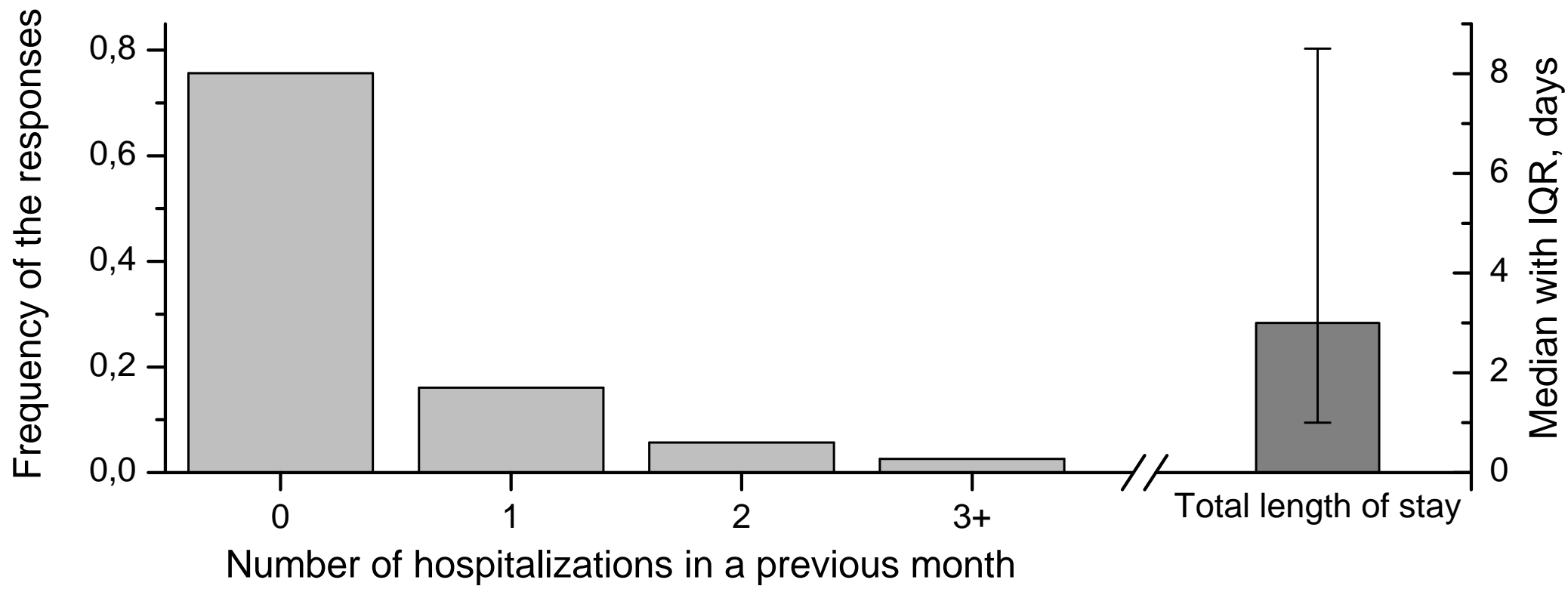

Supplement: S1 Fig — (PDF) [file pone.0168586.s002.pdf]

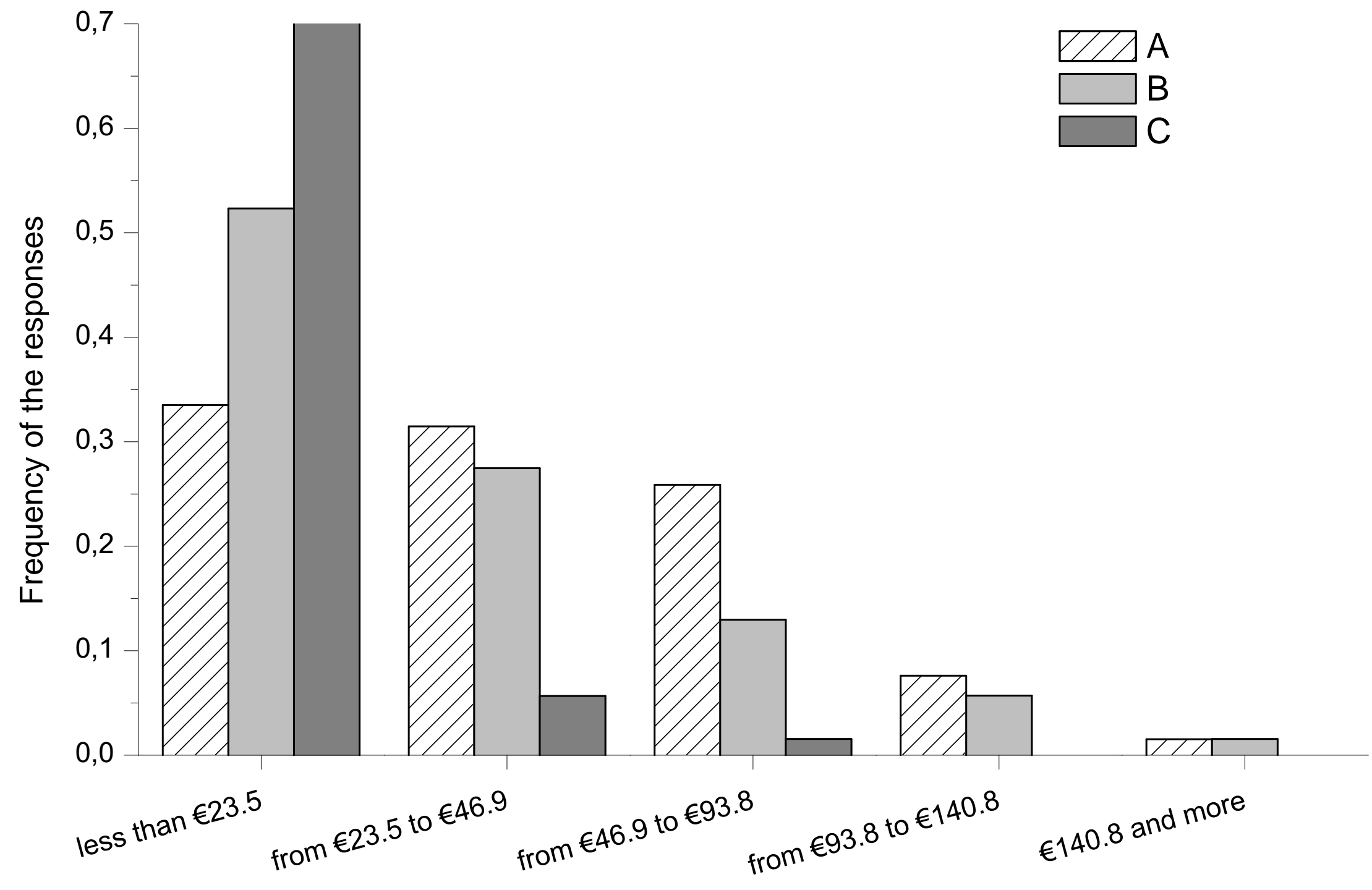

Supplement: S2 Fig — (A) Medications prescribed or recommended by physicians; (B) dietary supplements, special diet, special equipment, transportation to the medical facility; (C) informational materials about the disease, additional hygiene products and others. (PDF) [file pone.0168586.s003.pdf]
