# Supplementary material for: Health-Related Quality of Life Impairment and Indirect Cost of Crohn’s Disease: A Self-Report Study in Poland
Source: PLoS One. 2016 Dec 16;11(12):e0168586. doi: 10.1371/journal.pone.0168586 (PMC5161376; doi:10.1371/journal.pone.0168586)
Supplement: S1 Table — (PDF) [file pone.0168586.s004.pdf]

**Supplemental Information 2.** Additional **c**haracteristics of study participants by disease activity.

| **Characteristic** | **Subcategory** | **Remission (N=93)** | **Active disease (N=105)** | **All patients (N=200)** | **P value between subgroups** |
|---|---|---|---|---|---|
| Age, mean (SD) | | 30.60 (9.94) | 32.86 (10.75) | 31.80 (10.41) | 0.087 |
| Male gender, n (%) | | 40 (43.0) | 44 (41.9) | 84 (42.2) | 0.875 |
| Age at diagnosis, mean (SD) | | 24.14 (10.26) | 24.73 (10.88) | 24.46 (10.57) | 0.548 |
| Place of residence, n (%) | City with a population of ≥100 thousands | 56 (60.2) | 52 (50.0) | 110 (55.3) | 0.227 |
| | City with a population of <100 thousands | 19 (20.4) | 32 (30.8) | 51 (25.6) | |
| | Village | 18 (19,4) | 20 (19.2) | 38 (19.1) | |
| Comorbidities | Any, n (%) | 35 (37.6) | 50 (47.6) | 87 (43.5) | 0.157 |
| | Number of comorbidities [a], median (IQR) | 1 (1 to 2) | 1 (1 to 2) | 1 (1 to 2) | 0.666 |
| Comorbidities with >5% prevalence [a] | Allergies, n (% [a]) | 5 (14.3) | 7 (14.0) | 12 (13.8%) | 0.970 |
| | Psoriasis, n (% [a]) | 3 (8.6) | 7 (14.0) | 11 (12.6) | 0.445 |
| | Asthma, n (% [a]) | 4 (11.4) | 5 (10.0) | 9 (10.3) | 0.833 |
| | Hashimoto's thyroiditis, n (% [a]) | 2 (5.7) | 5 (10.0) | 7 (8.0) | 0.479 |
| | Celiac disease, n (% [a]) | 2 (5.7) | 3 (6.0) | 5 (5.7) | 0.956 |
| Penetrating disease course | Absent, n (%) | 66 (70.97) | 60 (58.82) | 126 (63.96) | 0.127 |
| | Present, n (%) | 19 (20.43) | 34 (33.33) | 54 (27.41) | |
| | 'don't know'/ 'don't remember', n (%) | 8 (8.60) | 8 (7.84) | 17 (8.63) | |
| Current treatment, n (%) | Mesalazine or sulfasalazine | 75 (80.6) | 90 (85.7) | 166 (83.0) | 0.339 |
| | Azathioprine, mercaptopurine or methotrexate | 52 (55.9) | 50 (47.6) | 104 (52.0) | 0.244 |
| | Budesonide, prednisone, prednisolone or methylprednisolone | 13 (14.0) | 37 (35.2) | 51 (25.5) | **0.001** |
| | Infliximab or adalimumab | 10 (10.8) | 18 (17.1) | 28 (14.0) | 0.198 |

| Characteristic | Subcategory | Remission (N=93) | Active disease (N=105) | All patients (N=200) | P value between subgroups |
|---|---|---|---|---|---|
| Surgical treatment in the past | Any, n (%) | 38 (40.9) | 53 (51.5) | 92 (46.5) | 0.138 |
| | Number of surgeries [a], median (IQR) | 2 (1 to 3) | 2 (1 to 3) | 2 (1 to 3) | 0.969 |
| | Months since last surgery [a], median (IQR) | 33 (12 to 70) | 27 (10.5 to 55) | 30 (12 to 60) | 0.781 |
| Disease activity at last clinical assessment, as indicated by responder | Remission or mild disease, n (%) | 84 (90.3) | 57 (54.3) | 141 (70.5) | **<0.001** |
| | Moderate or severe disease, n (%) | 6 (6.5) | 44 (41.9) | 52 (26.0) | |
| | 'don't know'/ 'don't remember', n (%) | 3 (3.2) | 4 (3.8) | 7 (3.5) | |
| Current disease activity | P-HBI, mean score (SD) | 1.88 (1.37) | 9.20 (3.98) | 5.76 (4.76) | not applicable |
| | Remission or mild disease, n (%) | 93 (100) | 41 (39.0) | 134 (67.7) | not applicable |
| | Moderate disease, n (%) | n/a | 58 (55.2) | 58 (29,3) | |
| | Severe disease, n (%) | n/a | 6 (5.7) | 6 (3.0) | |
| Current work status | Any occupational activity, n (%) | 60 (64.5) | 58 (55.2) | 120 (60.0) | 0.184 |
| | Full-time, n (% [a]) | 51 (85.0) | 47 (85.5) | 99 (84.6) | 0.982 |
| | Part-time, n (% [a]) | 3 (5.0) | 3 (5.5) | 6 (5.1) | |
| | Retired, n (%) | 2 (2.2) | 2 (1.9) | 4 (2.0) | 0.902 |
| | On a social or disability pension, n (%) | 4 (4.3) | 16 (15.2) | 21 (10.5) | **0.011** |
| | Student, n (%) | 21 (22.6) | 21 (20.0) | 42 (21.0) | 0.658 |

[a] among participants with higher-level category characteristic
